# Supplementary material for: Biphasic regulation of osteoblast development via the ERK MAPK–mTOR pathway
Source: eLife. 2022 Aug 17;11:e78069. doi: 10.7554/eLife.78069 (PMC9417416; doi:10.7554/eLife.78069)
Supplement: Figure 3—figure supplement 5—source data 1. [file elife-78069-fig3-figsupp5-data1.pdf]

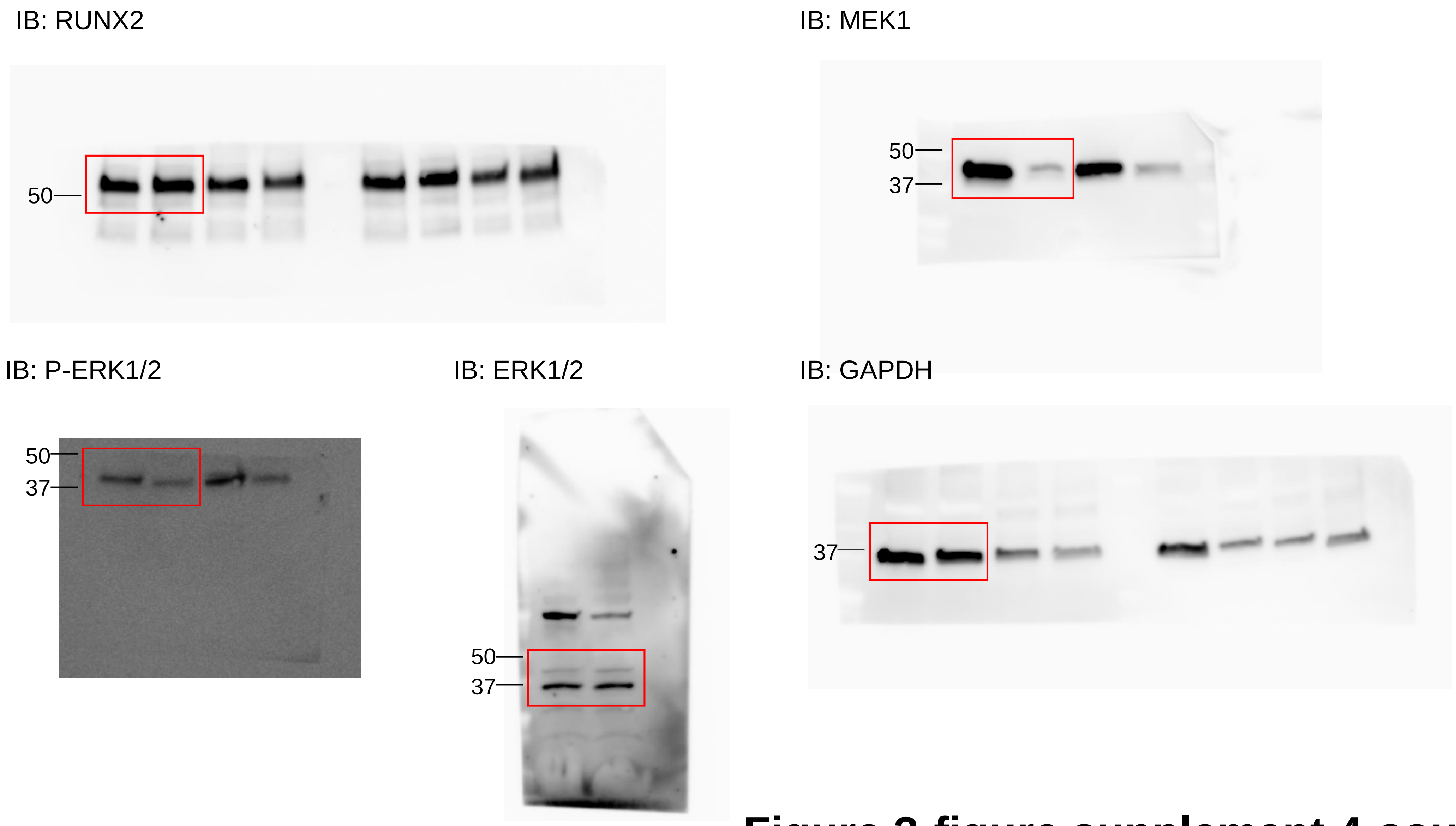

Figure 3-figure supplement 4-source data 1

Full immunoblots for Figure 3-figure supplement 4A
