# Supplementary material for: Biphasic regulation of osteoblast development via the ERK MAPK–mTOR pathway
Source: eLife. 2022 Aug 17;11:e78069. doi: 10.7554/eLife.78069 (PMC9417416; doi:10.7554/eLife.78069)
Supplement: Figure 5—figure supplement 1—source data 1. [file elife-78069-fig5-figsupp1-data1.pdf]

IB: P-AKT

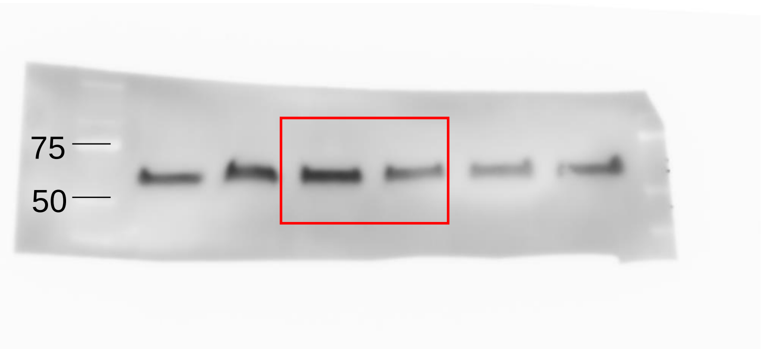

IB: P-SGK1

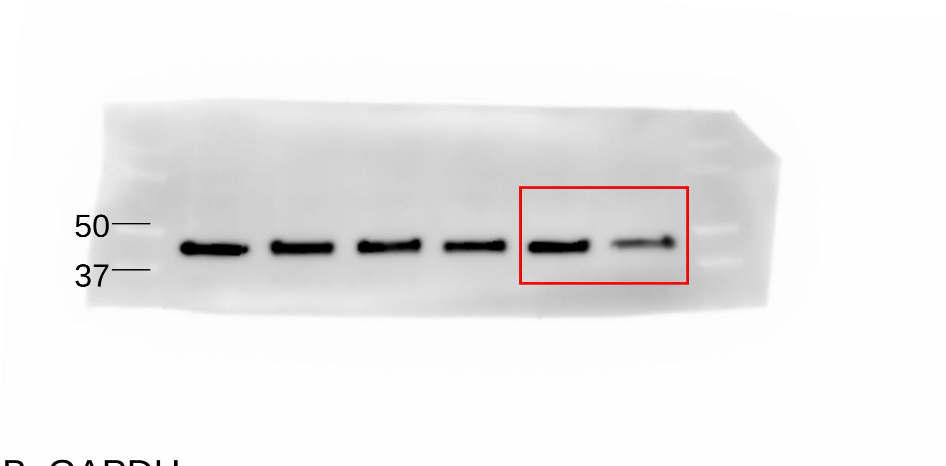

IB: P-p70S6K

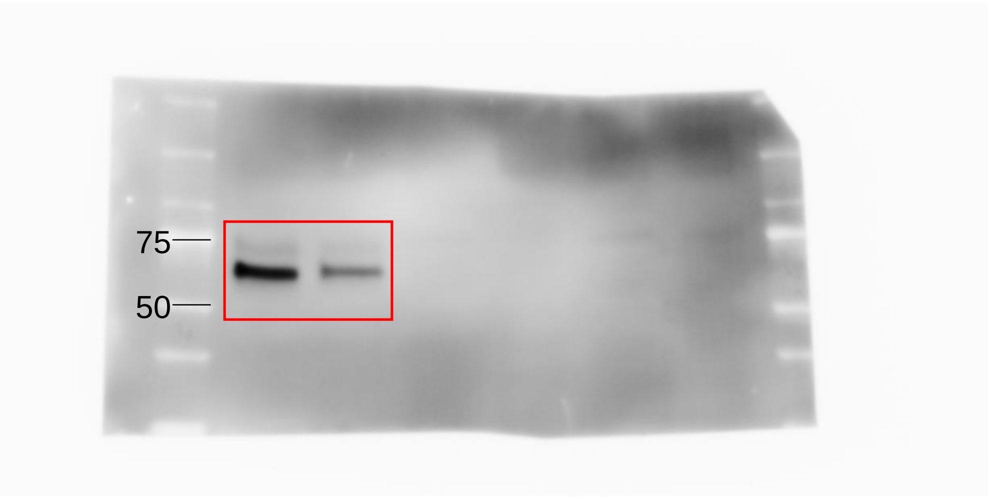

IB: GAPDH

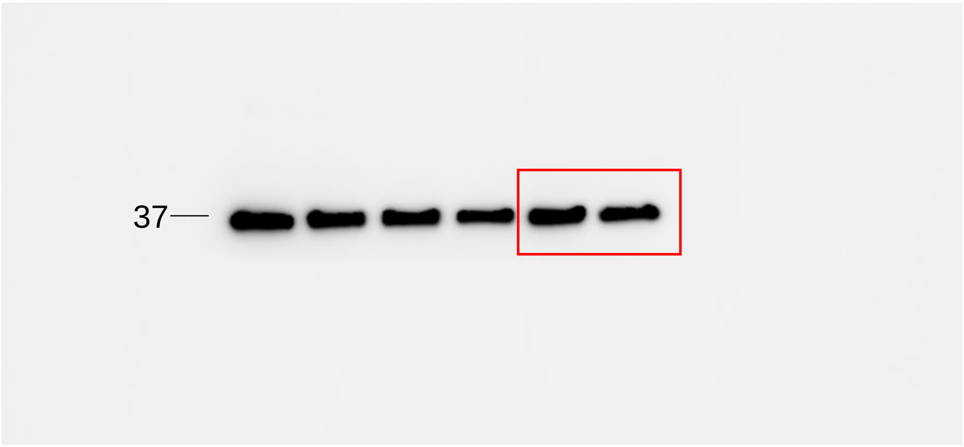

Figure 5-figure supplement 1-source data 1

Full immunoblots for Figure 5-figure supplement 1

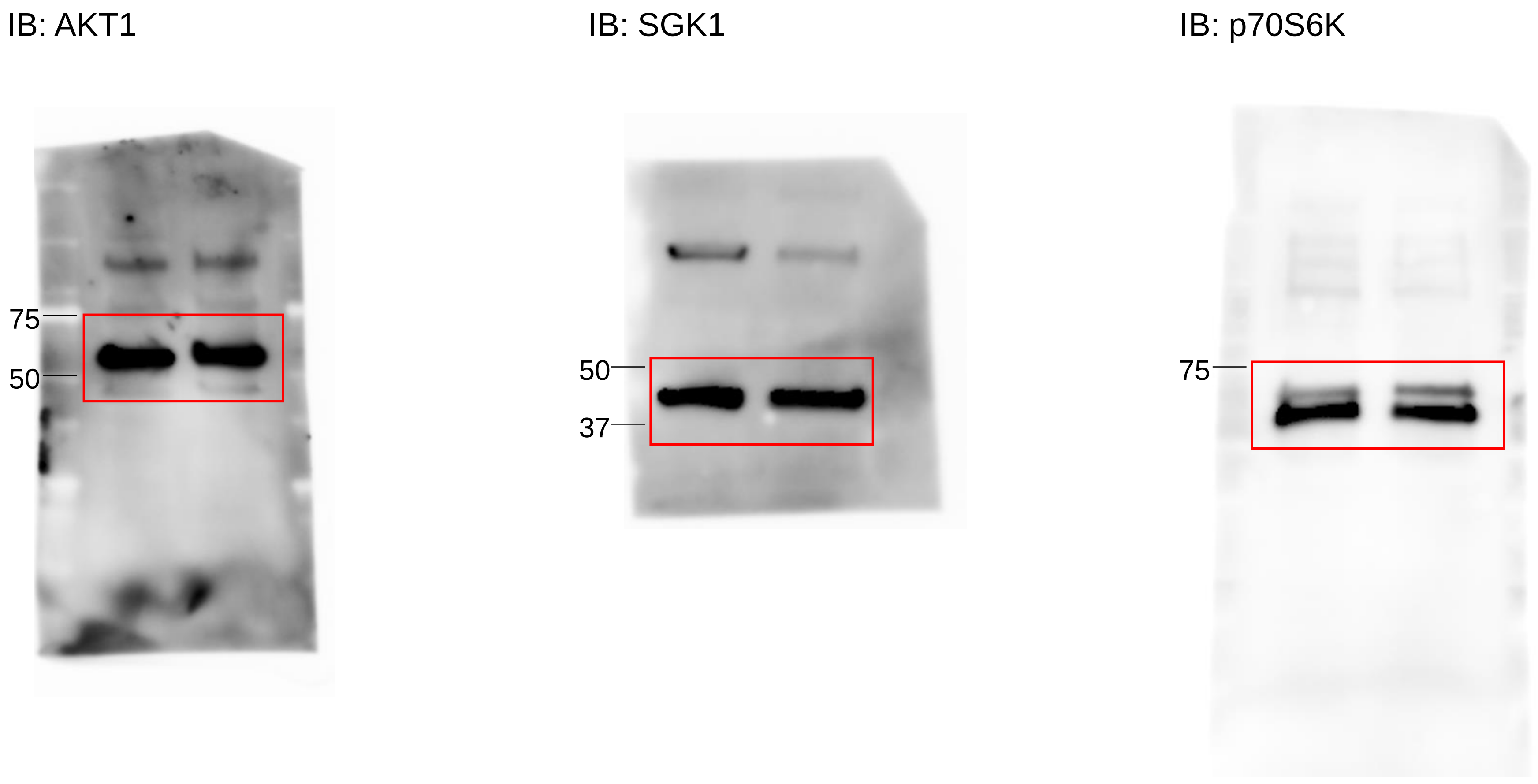

Figure 5-figure supplement 1-source data 1

Full immunoblots for Figure 5-figure supplement 1
