# Supplementary material for: Biphasic regulation of osteoblast development via the ERK MAPK–mTOR pathway
Source: eLife. 2022 Aug 17;11:e78069. doi: 10.7554/eLife.78069 (PMC9417416; doi:10.7554/eLife.78069)
Supplement: Figure 6—source data 1. [file elife-78069-fig6-data1.pdf]

IB: P-p70S6K

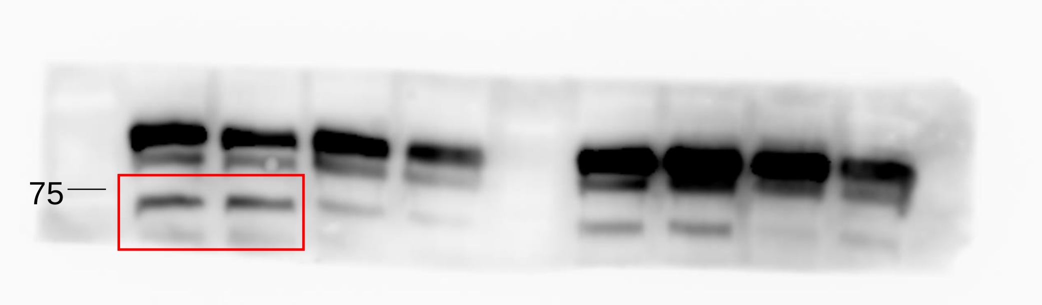

IB: P-4EBP1

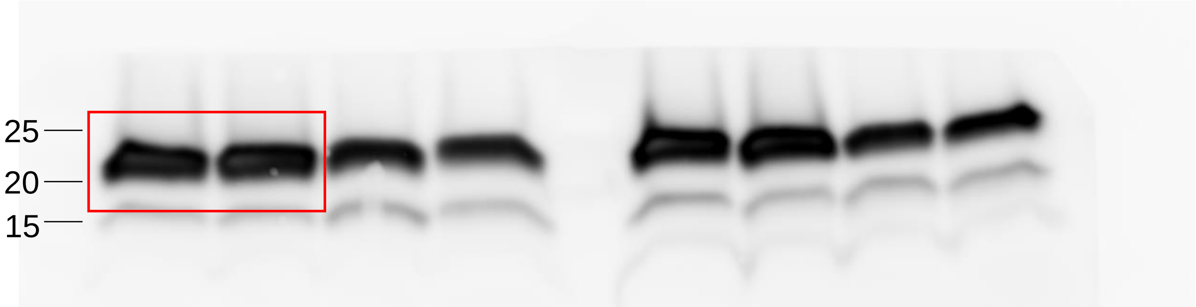

IB: P-ERK1/2

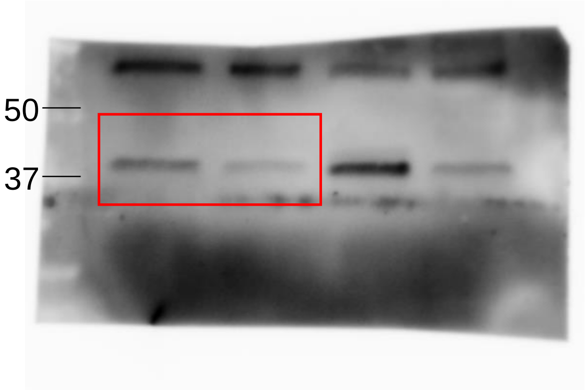

IB: MEK1

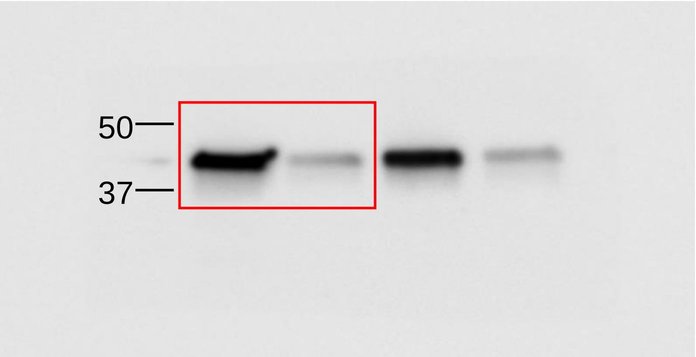

IB: GAPDH

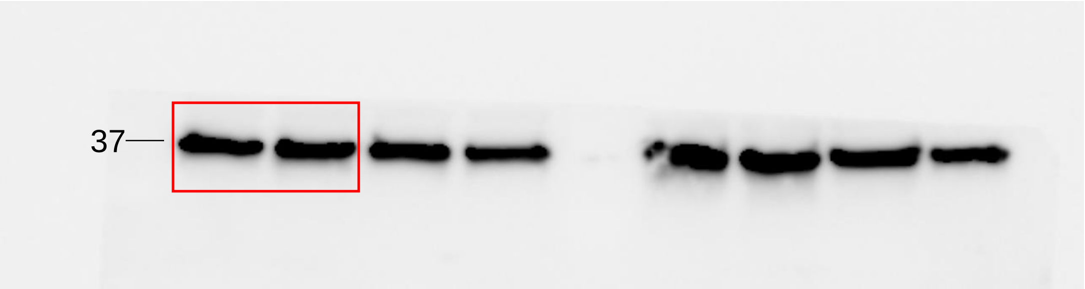

Figure 6-source data 1

Full immunoblots for Figure 6A (left)

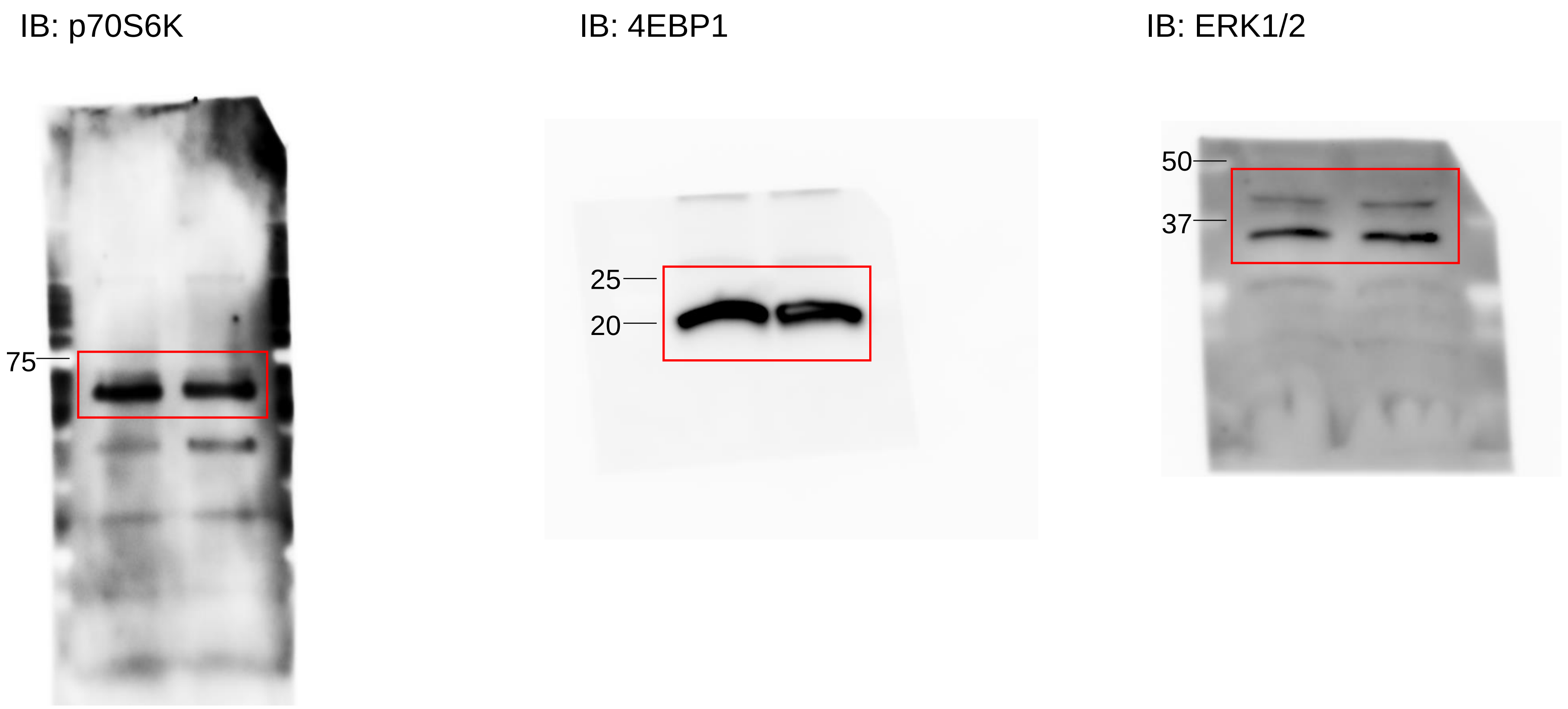

Figure 6-source data 1

Full immunoblots for Figure 6A (left)

IB: P-AKT

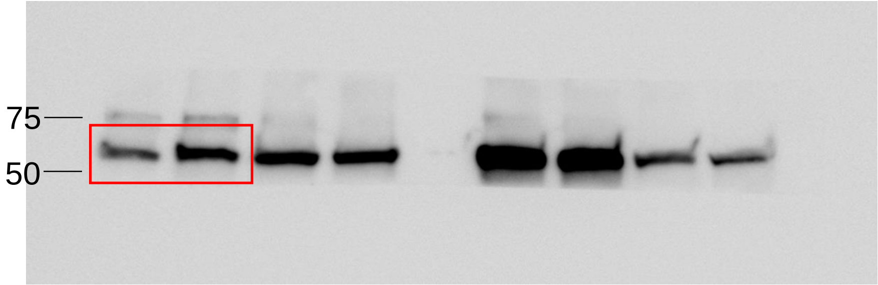

IB: P-SGK1

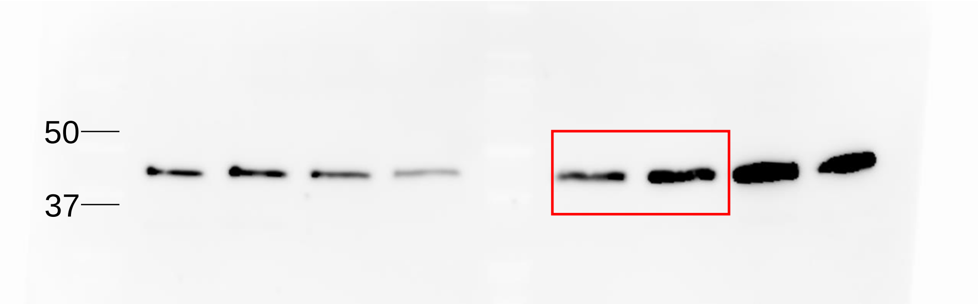

IB: P-NDRG1

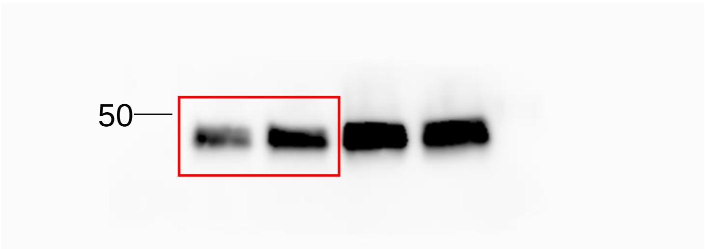

IB: GAPDH

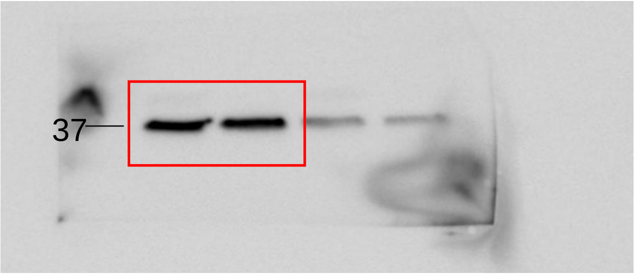

Figure 6-source data 1

Full immunoblots for Figure 6A (right)

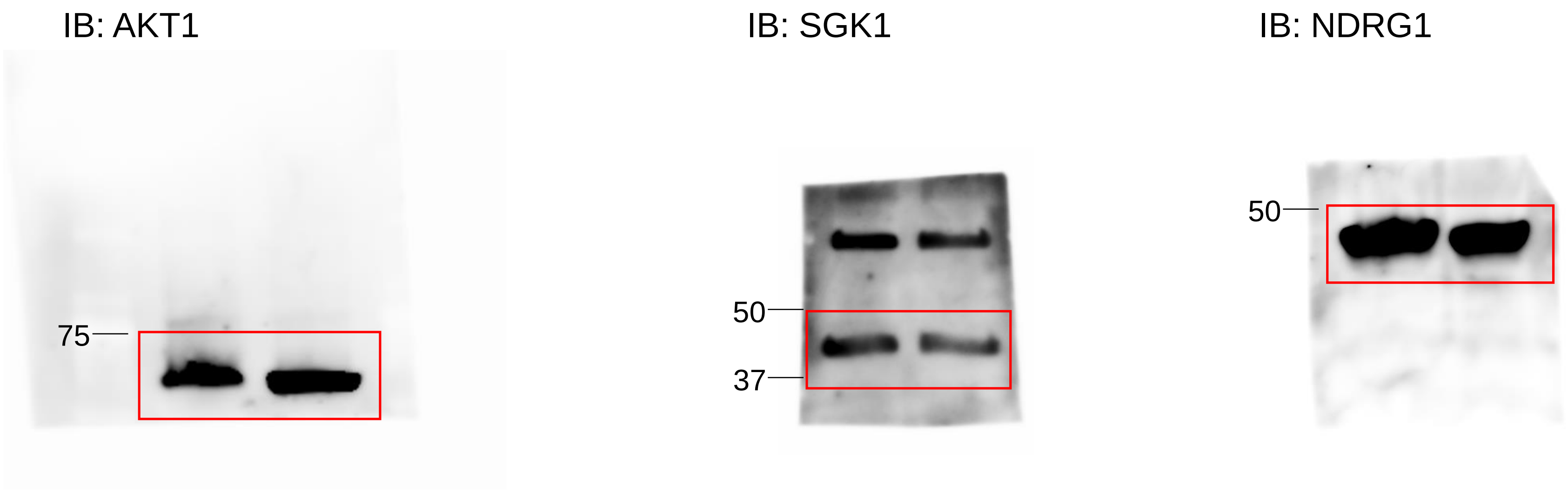

Figure 6-source data 1

Full immunoblots for Figure 6A (right)
